# CT imaging post-TAVI: Murphy’s first law in action—preparing to recognize the unexpected

**DOI:** 10.1186/s13244-024-01729-1

**Published:** 2024-06-20

**Authors:** Costanza Lisi, Federica Catapano, Federica Brilli, Vincenzo Scialò, Eleonora Corghi, Stefano Figliozzi, Ottavia Francesca Cozzi, Lorenzo Monti, Giulio Giuseppe Stefanini, Marco Francone

**Affiliations:** 1 Department of Biomedical Sciences, Humanitas University, via Rita Levi Montalcini 4, 20090 Milan, Pieve Emanuele Italy; 2 IRCCS Humanitas Research Hospital, via Manzoni 56, 20089 Milan, Rozzano Italy

**Keywords:** Cardiac imaging, Transfemoral aortic valve implantation (TAVI), Complications, Computed tomography angiography (CTA)

## Abstract

**Abstract:**

Transfemoral aortic valve implantation (TAVI) has been long considered the standard of therapy for high-risk patients with severe aortic-stenosis and is now effectively employed in place of surgical aortic valve replacement also in intermediate-risk patients. The potential lasting consequences of minor complications, which might have limited impact on elderly patients, could be more noteworthy in the longer term when occurring in younger individuals. That’s why a greater focus on early diagnosis, correct management, and prevention of post-procedural complications is key to achieve satisfactory results. ECG-triggered multidetector computed tomography angiography (CTA) is the mainstay imaging modality for pre-procedural planning of TAVI and is also used for post-interventional early detection of both acute and long-term complications. CTA allows detailed morphological analysis of the valve and its movement throughout the entire cardiac cycle. Moreover, stent position, coronary artery branches, and integrity of the aortic root can be precisely evaluated. Imaging reliability implies the correct technical setting of the computed tomography scan, knowledge of valve type, normal post-interventional findings, and awareness of classic and life-threatening complications after a TAVI procedure. This educational review discusses the main post-procedural complications of TAVI with a specific imaging focus, trying to clearly describe the technical aspects of CTA Imaging in post-TAVI and its clinical applications and challenges, with a final focus on future perspectives and emerging technologies.

**Critical relevance statement:**

This review undertakes an analysis of the role computed tomography angiography (CTA) plays in the assessment of post-TAVI complications. Highlighting the educational issues related to the topic, empowers radiologists to refine their clinical approach, contributing to enhanced patient care.

**Key Points:**

- Prompt recognition of TAVI complications, ranging from value issues to death, is crucial.
- Adherence to recommended scanning protocols, and the optimization of tailored protocols, is essential.
- CTA is central in the diagnosis of TAVI complications and functions as a gatekeeper to treatment.

**Graphical Abstract:**

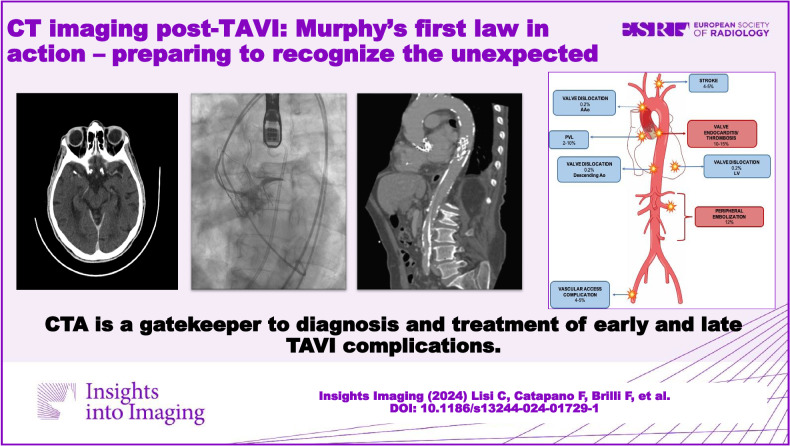

## Introduction

Transfemoral aortic valve implantation (TAVI) has been long considered the standard of therapy for high-risk patients with severe aortic-stenosis [1] and is now also effectively applied in intermediate-risk groups as an alternative to surgical aortic valve replacement (SAVR) also in intermediate-risk patients, as demonstrated by Surgical Replacement and Transcatheter Aortic Valve Implantation (SURTAVI) trial [2]. As the inclusion criteria for transfemoral aortic valve replacement are continuously expanding to lower-risk patients [3], the exponential increase in the number of procedures performed yearly makes radiologists frequently face post-procedural complications. Potential long-term impacts of ‘minor’ complications, that may have little effect on elderly patients, may be more significant in younger individuals. Therefore a greater focus on early diagnosis, correct management, and prevention of post-procedural complications is key to achieve satisfactory results [4]. ECG-triggered multidetector computed tomography angiography (CTA) is the mainstay imaging modality for pre-procedural planning of TAVI and is also used for post-interventional early detection of both acute and long-term complications [5]. In the clinical routine, valve function is evaluated by means of transthoracic echocardiography (TTE). However, the valve leaflets often cannot be directly visualized on echocardiography. CTA allows detailed morphological analysis of the valve and its motion throughout the entire cardiac cycle. Moreover, stent position, coronary artery branches, and integrity of the aortic root can be precisely evaluated. Imaging reliability implies the correct technical setting of the computed tomography (CT) scan, knowledge of the transcatheter valve model, normal post-interventional findings, and awareness of classic and life-threatening complications after a TAVI procedure [6]. This educational review discusses the main post-procedural complications of TAVI with a specific imaging focus, trying to clearly describe the technical aspects of CTA Imaging in post-TAVI and its clinical applications and challenges with a final focus on future perspectives and emerging technologies.

## Technical aspects of CT imaging in post-TAVI complications

CTA significantly enhances the management of post-TAVI complications, offering accessibility, cost-effectiveness, and detailed anatomical insights for optimal medical or surgical treatment [7].

According to the latest international consensus on TAVI imaging [8, 9], the minimum technological equipment required for peri-procedural imaging consists of a 64-slice or dual-source scanner. This requirement ensures sufficient temporal and spatial resolution while maintaining an acceptable total effective radiation dose.

Indeed, when imaging complications, adherence to the well-established pre-TAVI imaging acquisition protocol is essential, but a patient- and diagnostic question-tailored approach is required to reduce contrast media and radiation dose administration.

An ECG-gated (spiral or sequential) scan of the chest or the heart before and after contrast administration is mandatory to evaluate the position and expansion of the valve, as well as to look for potential perivalvular/periaortic complications and/or coronary assessment. Retrospective ECG-gating should be preferred to allow dynamic evaluation of the valve, using a multiphase or, at a minimum, biphasic (optimal diastole and systole) reconstructions [9, 10].

A slice thickness of 1.0 mm or lower is required for images to be appropriately reconstructed, enabling precise multiplanar reformations [8].

In case of access-site or access-related complications, the protocol includes an ungated spiral scan encompassing both the subclavian and femoral arteries [7]. Additionally, a triphasic protocol is employed when there is suspicion of vascular dissection, pseudoaneurysm, or rupture [11].

Deciding the study protocol based on the suspected complication aligns with the ALARA (as low as reasonably achievable) principle and helps customize contrast administration dose. Acute kidney injury is a well-known complication in elderly patients, increasingly associated with higher mortality rates [12]. With more advanced technologies, low-dose and ultra-low-dose contrast administration protocols have been successfully proposed in these patients [13]. Acquisition protocol with 64/128 slice CT system includes a retrospectively gated scan of the aortic root followed by a non-ECG-gated helical scan up the aortic arch, down the bifurcation of common femoral arteries, with a kVp variable from 100 to 140 according to patient’s body mass index, and iodinated contrast volume of approximatively 90–100 mL [8, 14, 15]. The major drawback of these scanners is the limited cranio-caudal coverage without gantry movement (typically only 20–40 mm). To overcome this drawback, wide-field detectors have been developed that allow coverage of more than 40 mm per rotation.

Wide detector volume CT scanners allow a single volumetric whole-heart acquisition during the complete heart cycle with a slice thickness of 0.625 mm, reduced dose delivered (kVp of 100–120 and 500–700 mAs) and contrast administration (up to a minimum of 38 mL) [16]. The following non-gated spiral scan can be performed with a delay of about 3 s [8, 17].

Moreover, iterative reconstructions can be used to minimize the cone-beam artifacts of the prosthesis and calcifications and reduce contrast volume with low tube potential [18, 19].

While dual-source scanners with non-high-pitch mode utilize a similar scanning protocol as 64/128 slices systems but with lower mAs and improved reconstruction filters, third-generation dual-source CT scanners offer the capability of high-pitch CTA of the entire aorta with a retrospectively ECG-gated scan mode or two acquisitions with a fast scan coverage [18, 20]. This advanced technology provides major advantages for these patients, enabling a short overall acquisition time and minimizing contrast exposure [21].

The last-generation scanner is the photon-counting detector CT (PCD-CT), equipped with a semiconductor detector (e.g., gadolinium-oxide or gadolinium-oxysulfide), which is more geometrically efficient than the conventional scintillation detector, enabling ultra-high resolution and simultaneous spectral imaging [22].

In their case series, Van Der Bie et al [23] showed the potential advantages of this system in post-procedural imaging. PCD-CT enables the detection of even small hypoattenuating leaflet thickening (HALT) and allows for the evaluation of iodine uptake in the leaflets through multiple spectral reconstructions. Reduction of beam hardening and blooming artifacts allows accurate imaging of stent structure and facilitates assessment of valve positioning and leaflet motion [23].

Thus, this technology could lead to advanced applications, enabling early detection of complications like valve failure, paravalvular leakage (PVL), and endocarditis, while minimizing the contrast dose, a major concern when imaging this specific patient population (Table 1).

**Table 1:** CTA imaging protocol acquisition for pre- and post-TAVI evaluation

Gating	ECG-gating Retrospective (functional evaluation over the cardiac cycle) Prospective (coronary evaluation)
Contrast media	Iodinated contrast media: 1/1.5 mL/kg Bi-phasic injection: 5 mL/s followed by 40/50 mL saline (5 mL/s)
Acquisition	Bolus tracking: ROI in ascending aorta (100 HU threshold)
Scan coverage	Aortic arch-diaphragm
Tube current and kVp	Automated current modulation 100–140 kVp
Image reconstruction	1.0 mm or lower slice thickness FBP, iterative reconstruction

*FBP* filtered-back-projections

In the clinical suspicion of peri-procedural TAVI hyperacute stroke, a dedicated brain CT is mandatory. The full protocol includes non-enhanced CT, CTA, and perfusion-CT, to optimize patient outcomes in this scenario [24]. Non-enhanced CT rules out hemorrhage or ischemia mimics and helps in identifying calcific emboli. CTA shows whether an obstruction in a major vessel is present, and perfusion-CT differentiates the ischemic core from the ischemic penumbra [25]. CTA consists of a thin-section volumetric CT examination performed with a time-optimized bolus of non-ionic contrast medium. Acquisition volume includes from the aortic arch to the vertex with thin slice thickness and small pitch. The bolus tracking technique is used with ROI on the ascending aorta. CTA aims at the main intracranial vessel evaluation with multiplanar maximum-intensity projection (MIP) reformatting on a three-dimensional workstation. It identifies the occlusion site, demonstrates vessel dissection, and grades collateral flow, to guide interventional treatment [26]. Perfusion-CT is performed with a separate contrast medium bolus at a high flow rate (4–5 mL/s) to monitor its first pass throughout the cerebral circulation. Continuous cine imaging is conducted over the same tissue slab during the dynamic contrast administration, to get perfusion parameters aiming at differentiating the ischemic core from the penumbra [27]. The whole examination can be undertaken and analyzed within around 15 min with the new-generation multidetector CT scanners.

## Imaging assessment of complications

**Fig. 1:**
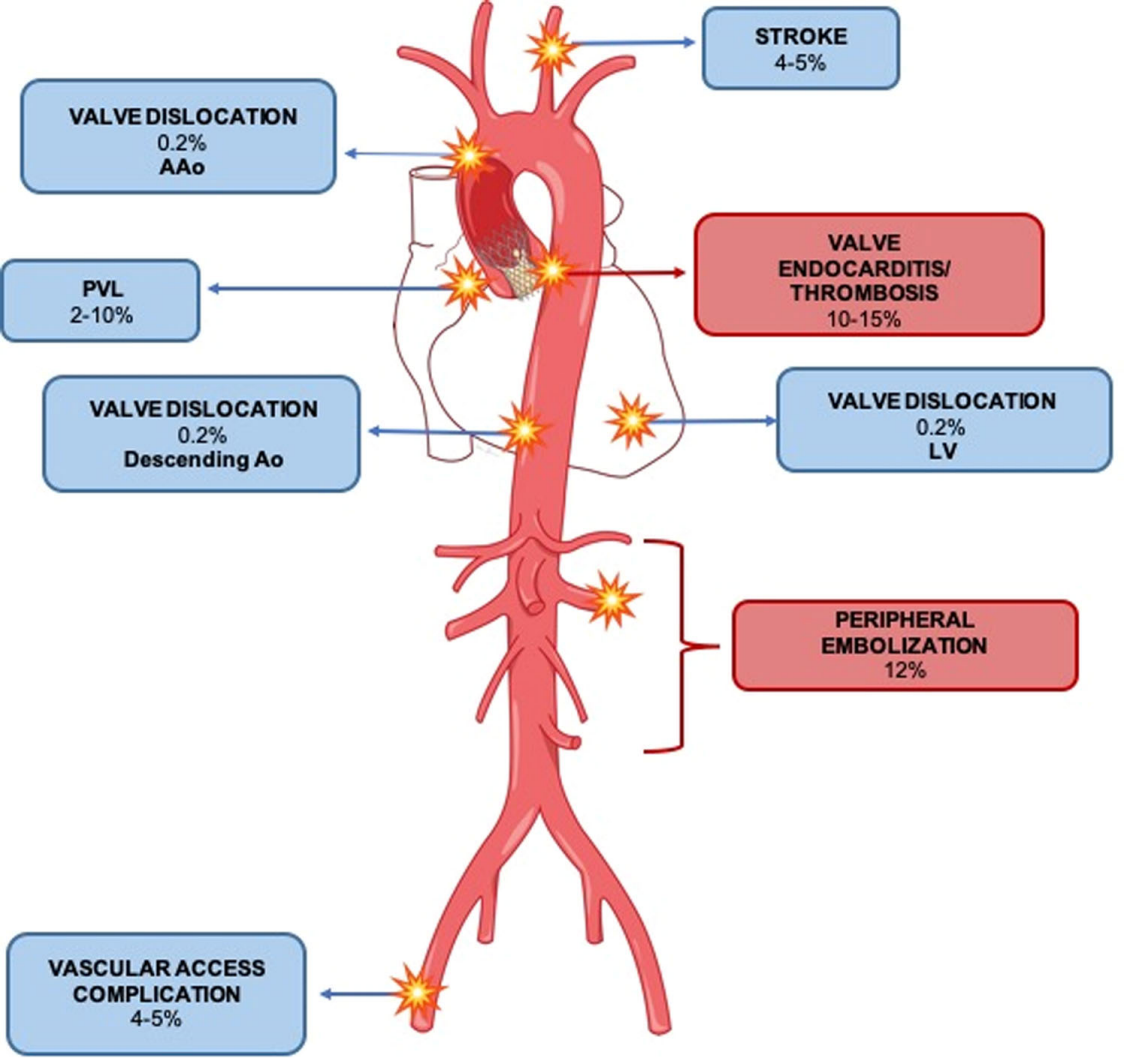
Schematic representation of main post-TAVI complications identified by CTA and their incidence. Blue square for peri-procedural complications, red square for late complications

When dealing with post-TAVI complications, radiologists must be able to perform the correct differential diagnosis, focusing on the most common complications related to the procedure timing. Some of them may occur during in-hospital stay immediately after the valve replacement and others are mainly encountered after patient discharge and during long-term follow-up [28]. This article focuses on complications diagnosed through CTA and their typical imaging features, distinguishing between peri-procedural issues—specifically those related to valve position, function, and vascular access—and late follow-up problems such as paravalvular leaks, structural valve deterioration, and endocarditis (Fig. 1).

### Evaluation of valve position and function

**Fig. 2:**
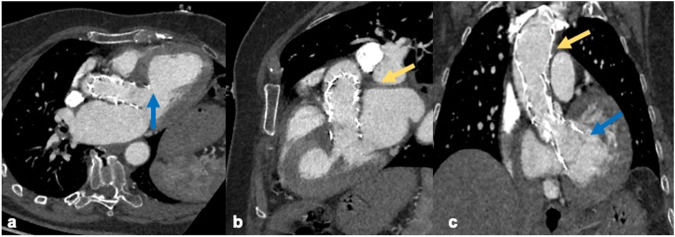
MPR of a infaranular valve dislocation (**a**, **b**, **c**). Transcatheter sel-expandable aortic valve (Portico) dislocated to the left ventricular outflow tract (**a**, **c**, blue arrow) with anterior mitral valve leaflet impairment (**a**). After a ViV procedure, a second prosthetic module was correctly positioned (**c**, yellow arrow)

**Fig. 3:**
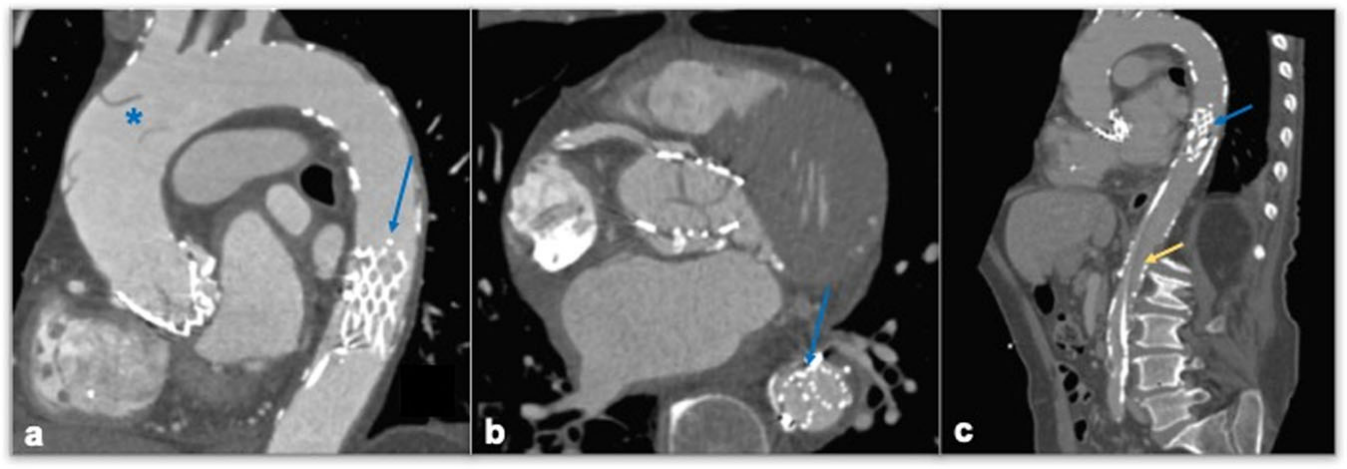
A 55-year-old patient CTA control after TAVI positioning for prosthesis dislocation. CTA images show, aortic prosthesis dislocation in the descending aorta (**a**, **b**, **c** blue arrow), causing focal ascending aorta dissection (blue asterisk). Sagittal MPR also shows the presence of a vascular catheter inside the thoracoabdominal aorta, trapped in the dislocated prosthesis (**c**, yellow arrow)

**Fig. 4:**
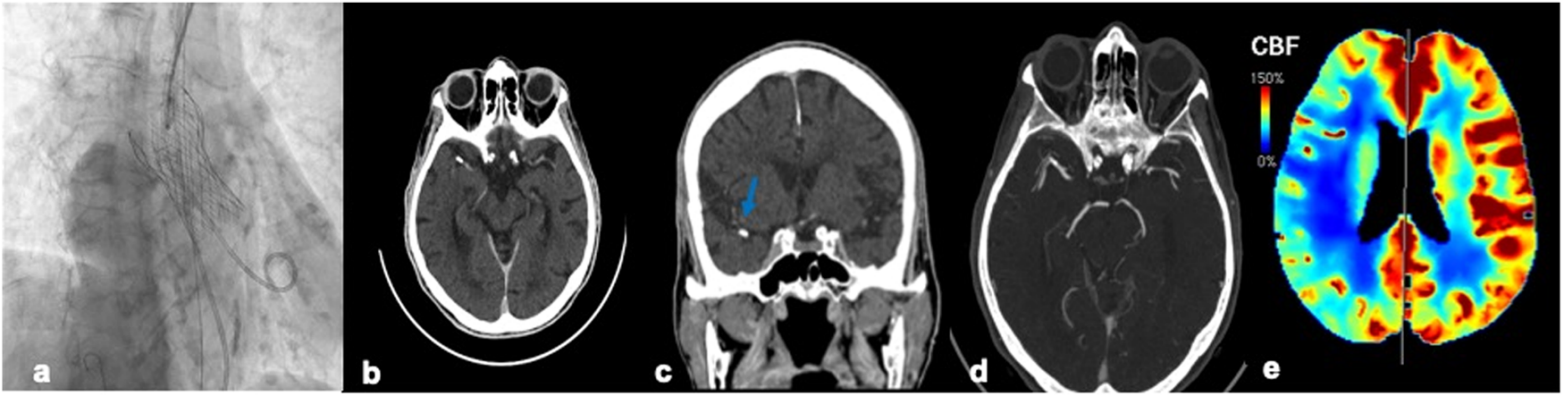
An 83-year-old patient presenting to the emergency room with dysarthria, difficulty in walking, and dropping lip 7 days after TAVI for severely calcific aortic-stenosis (**a**). A brain CTA was performed, demonstrating a calcific embolus in the right middle cerebral artery (**b**, **c** arrow), determining incomplete occlusion of the vessel (**d**, arrow), as a result of distant calcific embolization during TAVI. Perfusion-CT shows an area of ischemic penumbra in the right cerebral hemisphere, with reduced cerebral blood flow (CBF) (**e**). The patient was treated with a thrombectomy and got a resolution of clinical symptoms

**Fig. 5:**
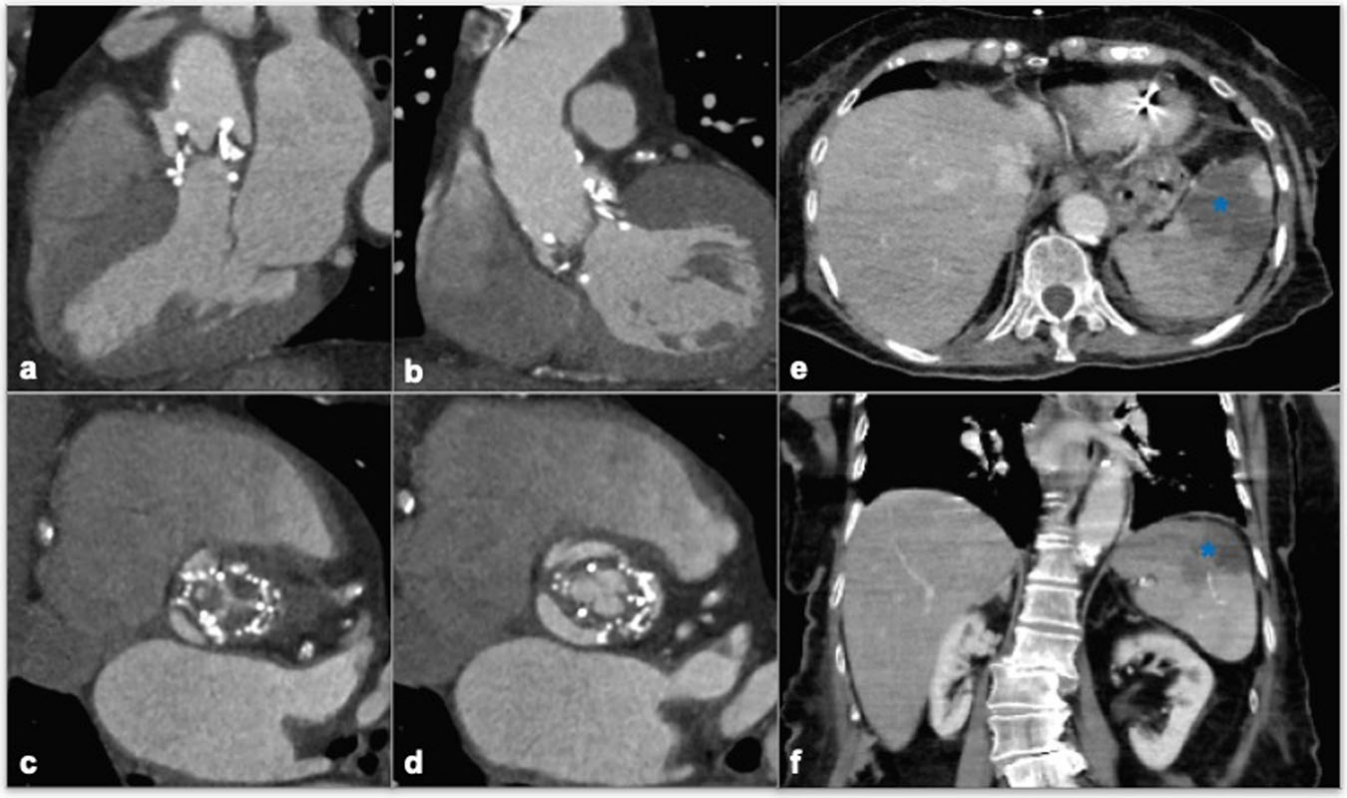
An 85-year-old woman, subjected to TAVI, developing fever and abdominal pain with elevated white blood cell count and C-reactive protein. CCTA is performed in the suspicion of endocarditis and also extended to the abdomen to rule out peripheral embolization. CCTA para-axial, coronal, and sagittal vie (**a**, **b**, **c**, **d**) show thick ipodense thrombotic apposition over prosthesis leaflets (**b**, **c** blue arrow). Abdominal CT also shows splenic infarction due to septic embolization (**e**, **f** asterisk). Blood culture turns positive for *Enterococcus faecium*

Prosthetic valve correct positioning is central to grant satisfactory post-operative results and favorable patients’ prognosis. CTA is known to exert a central role in evaluating it [28]. Several valve types can be used for TAVI, giving the opportunity to tailor valve deployment according to patients’ anatomical characteristics; depending on prosthesis design, a specific correct positioning exists, which is usually assessed during the interventional procedure, but can also be verified at post-procedural CTA when necessary [29] (Fig. 2). Three main valve types are routinely implanted, namely the Edwards SAPIEN, the CoreValve and Lotus prosthesis, according to different deployment methods (Table 2). In the case of balloon-expandable prosthesis, like the Edwards SAPIEN, the stent is made to fully expand circularly to squeeze the calcific valve against the aortic root. In some cases, massive calcifications may prevent the stent circumference from lying over the left ventricular outflow tract (LVOT). As a rule, the prosthesis should be placed at the level or below the original leaflets, so that the mid-portion of the stent is coincident with the annulus, with half stent extending proximally and half distally. Despite the correct positioning, the prosthesis frame may cover the coronary ostia, mainly the left one, but the Edwards SAPIEN valve is only sealed in its distal 2/3 so that coronary occlusion rarely occurs. Anyway, the radiologist should check for normal coronary ostia enhancement. Self-expandable prostheses, like CoreValve, expand automatically and they tend to adapt to the annulus shape, rather than being circular like Edwards SAPIEN. Its correct positioning is with the lower end of the vale 4 to 6 mm below the original annulus. A too-low position is defined when the distal end is 12 mm below it, while a too-high deployment happens when the lower end of the valve is above the annulus. Lotus valve, instead, is released via a three-arm mechanism, leading to a controlled expansion and possible retrieval and repositioning. Its correct positioning is at the level of the sinus of Valsalva, 5 mm distal to the annulus [30]. Coronary artery ostia obstruction is quite rare (total incidence is 0.66%) with all kinds of prostheses, as most devices are designed to preserve them. Ostia occlusion is commonly caused by native calcified valves, coronary dissection, and calcium embolization but CTA is almost never performed for this indication. Acute obstruction is treated via percutaneous transluminal coronary angioplasty and a protective catheterization is usually performed before inserting the valve to avoid this complication [31]. It’s nowadays well-known that the anatomical relationship between the transcatheter valves and coronary ostia may enhance the difficulty of getting interventional access to coronary ostia after TAVI [32]. Recent evidence suggests post-TAVI CTA may help in identifying “high-risk coronary access features”, helping interventional cardiologists to guide procedures. Unfavorable coronary access is encountered, as defined by Ochiai et al [33] when the coronary ostium is located below the skirt or in front of the transcatheter valve commissural posts above the skirt. They demonstrated this feature was significantly associated with the lower success rate of selective coronary engagement and, as such, it must be considered by radiologists whenever reporting CTA performed prior to invasive coronary angioplasty in post-TAVI patients. Incorrect positioning can also lead to stent dislocation (0.2% of patients), which needs prompt recognition and immediate surgical management, making CTA the gold standard imaging modality for diagnosis. The most common sites of migration are the ascending aorta and left ventricle (LV) (Fig. 3). Ventricular stent migration is usually due to high radial force during deployment; cranial migration into the aorta, instead, may be the consequence of malposition, excessively calcified leaflets, undersized prosthesis, and angulated aorta. Valve migration may also lead to embolization, with life-threatening blood flow obstruction to vital organs, like the brain, possibly leading to stroke or peripheral organs, determining ischemia [34] (Figs. 4 and  5).

**Table 2:** Schematic representation of different valve types and correct positioning parameters

Valve type	Correct positioning
EdwardSAPIEN (balloon-expandable)	Mid-portion of the stent coincident with the annulus
CoreValve (automatically expandable)	Lower end of the vale 4/6 mm below the original annulus
Lotus valve (mechanically expandable)	At the level of the sinus of Valsalva, 5 mm distal to the annulus

### Detection and characterization of paravalvular leaks and structural valve degeneration

PVL is a relatively common post-TAVI complication, with an incidence ranging from 2–10%. When associated with high-grade aortic regurgitation (AR), PVL increases patients’ mortality by inducing LV volume overload and consequent heart failure (HF), since left ventricular walls cannot bear an incremental end-diastolic pressure, with important functional consequences. It usually develops because of a triangular gap between the prosthesis and the native annulus, possibly due to annular-prosthesis size mismatch, heavy calcifications of native leaflets, and bicuspid aortic valve [35]. For this reason, accurate pre-TAVI CTA evaluation is necessary to prevent and predict PVL incidence. While echocardiography (either TTE or TEE) remains the primary imaging modality for detecting PVL during or after the procedure, CT represents more than an alternative for patients with poor acoustic windows. The primary goal of imaging the PVL is to precisely define the leak’s characteristics—such as its size, extent, position, and relation to the valve leaflets—and to rule out any concurrent issues like active endocarditis. While not routinely necessary, CT can offer a more in-depth anatomical assessment, aiding in the understanding of PVL mechanisms, dimensions and orientation, valve positioning, and the extent of calcification.

**Fig. 6:**
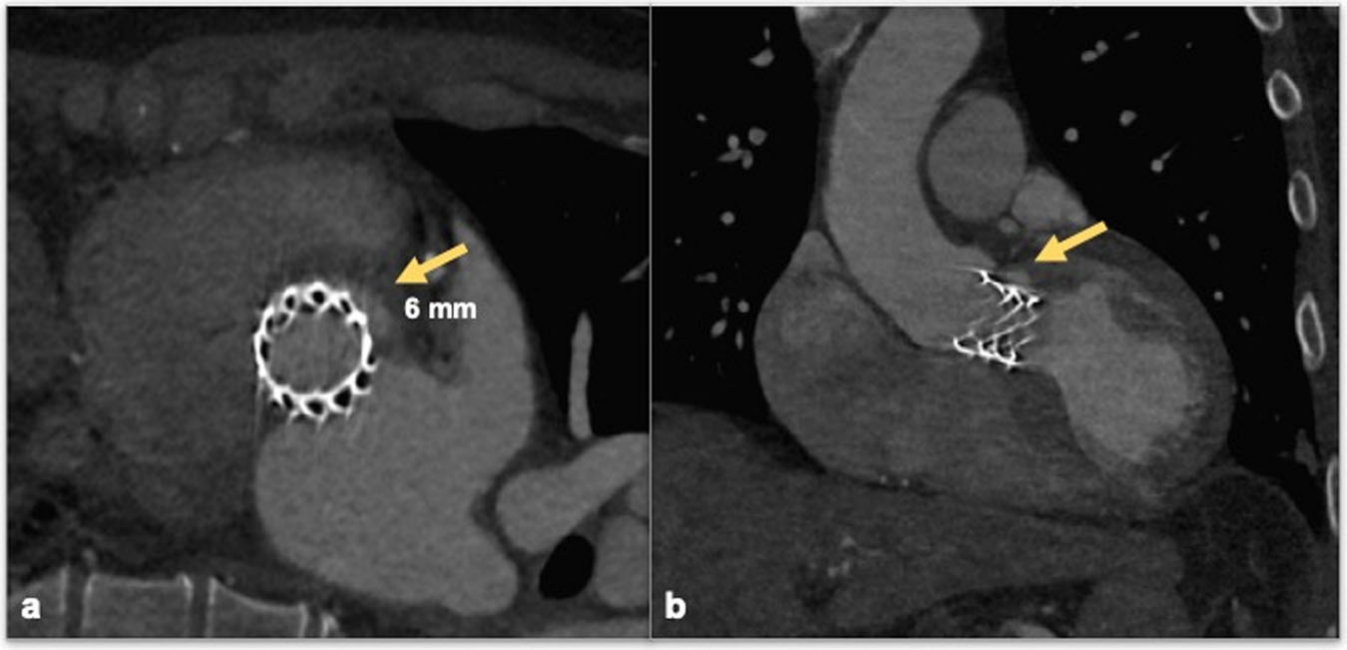
A 68-year-old male patient with severe aortic-stenosis, treated with Edwards SAPIENS aortic bioprosthesis, undergoing ECG-gated CTA for a TTE suspicion of the periprosthetic leak. CTA confirms a thin (6 mm thickness, arrow) PVL located just below the ostium of the main trunk in the axial oblique (**a**, arrow) and coronal plane (**b**, arrow)

PVL must be evaluated on a true axial plane and oblique reconstructions because pure coronal and sagittal images can hide crescent-shaped leaks. Radiologists should define the position of PVL with respect to the prosthetic valve in a clock-face orientation according to the surgical view (Fig. 6). Leakage must be differentiated from sutures and prosthetic material, which are clearly recognized in non-contrast images and have higher density compared to contrast medium in contrast-enhanced ones. Beam hardening artifacts close to the point where the prosthetic leaflet connects to the valve ring may also represent a mimicker of PVL, for this reason, iterative reconstructions and soft-tissue windows usually help to perform a correct evaluation [36]. PVL severity can be further assessed with multiple quantitative and also invasive echocardiographic and angiographic parameters, like RJW, vena-contracta in TE-echocardiography, 3-degrees Sellers opacification-criteria in Angiography, and AR index in Hemodynamics [37].

Structural valve degeneration (SVD) includes several pathological entities resulting in a permanent valve dysfunction, ranging from valve calcifications, tear, and retraction, causing stenosis to stent fractures, leaflet flail, and/or irregular motion resulting in regurgitation [38]. Since TAVI indication has been extended also to younger patients at intermediate risk, it is necessary to understand the mechanisms that determine SVD and affect prosthesis durability. Despite TTE being the gold standard imaging modality to detect it and evaluate trans-valvular gradients or regurgitation fraction, CTA should be considered as an additional modality to assess valve anatomy [36]. Due to its superior spatial resolution, CTA can be primarily used to identify leaflet thickening and/or calcifications [39]. SVD may manifest within a few years after TAVI and this is usually linked to chronic prosthesis inflammation due to several antigens, diabetes, chronic renal failure, hypertension, dyslipidemia, endocarditis, and hyperparathyroidism. A correlation between small prosthesis diameter and early tissue degeneration seems to exist, as a trigger of high-gradient stressed mechanisms over the valve. Anyway, in spite of the mechanisms leading to SVD and deterioration, a 5 years follow-up post-TAVI demonstrates patients develop a significant increase in mean gradient and central AR, but no reduction in left ventricular ejection fraction, meaning SVD does not cause hemodynamic impairment [40]. Hybrid imaging, particularly 18F-sodium fluoride (18F-NaF) PET/CT, is emerging as an effective method to detect SVD and thrombosis in patients after TAVI [41]. 18F-NaF uptake can identify active tissue calcification and may allow early identification of patients at higher risk of degeneration [42, 43]. However, the appropriate use and clinical implications of these imaging techniques are still an ongoing discussion.

As TAVI indication is expanding to younger and lower-risk patients, given their longer life expectancy, the increasing prevalence of SVD prompts us to find valid re-treatment options [44]. Redo-TAVI plays an increasingly relevant role in this context as an alternative to SAVR. Recent literature evidence shows no significant difference in 1-year mortality, stroke, pacemaker implantation, myocardial infarction, major vascular complications, and PVL [45]. Furthermore, the valve-in-valve (ViV) procedure is associated with low in-hospital mortality (1.25%) and good success rate (86.8%) [46]. Also in this context, CTA is gaining importance and is now considered an integral part of the pre-procedural planning for redo-TAVI. Pre-ViV CTA should be reported considering the following aspects [47]:

- motion and blooming artifacts should be corrected to avoid wrong measurements;
- native transcatheter valve must be analyzed looking for calcification presence and distribution, calcified native leaflets and sino-tubular-junction (STJ), coronary height, and dilated left ventricular outflow tract.
- inflow diameter, middle part, and outflow diameter of the first prosthesis must be measured and compared to the native valve to plan the eventual first transcatheter valve expansion.
- neoskirt size should be inferred from the first prosthesis leaflets height and ViV implantation depth
- alignment between coronary ostia and old prosthesis leaflets must be evaluated
- coronary ostia height with respect to the first prosthesis stent height should be measured: whenever the stent is below the coronary ostium, measure valve-to-coronary distance, while if the stent frame is above the coronary ostium, measure valve-to-STJ.

### Valve thrombosis and endocarditis

Although clinically significant valve thrombosis after TAVI is rare, subclinical valve thrombosis is the most commonly observed complication of post-interventional CTA (10%–15% incidence). Subclinical leaflet thrombosis, which can be observed as HALT, has been hypothesized as the underlying cause of reduced leaflet motion. These conditions might accelerate SVD and potentially act as a source of thromboembolic events [39, 48]. CT can be used to assess leaflet anatomy and motion, using thin-thickness multi-planar reconstructions (MPRs) in dynamic images of the prosthetic valve throughout the cardiac cycle. Prosthesis leaflets are very thin, typically thinner than surgical valves ( < 0.35 mm), and may only be visible on CTA as fine hypodense lines. HALT has been defined as one or more leaflets with hypo-attenuated thickening in at least two different MPR projections [49, 50] in at least two phases of the cardiac cycle [34] (Fig. 6).

Both TTE and trans-esophageal (TEE) are less sensitive than CTA in detecting valve thrombosis. They are roughly suitable to detect valve motion limitations, leaflet thickening, or increased flow velocity across the valve [30]. Infective endocarditis (IE) is a relatively rare condition after TAVI, with an incidence of 0.2%–3.1% at 1 year, but is associated with high mortality and complications, among which HF is the most common. Clinical suspicion, microbiological correlation, and additional imaging are required (modified Duke criteria) for the diagnosis. Echocardiography is the first-line imaging approach (50% sensitivity), identifying small mobile echogenic mass upon the cardiac surfaces or new onset valvular regurgitation. Echocardiographic diagnosis is challenging, especially due to blooming and beam hardening artifacts induced by prosthesis metal. CTA identifies IE as the thickening of prosthesis leaflets, similar to valve thrombosis, with free-floating vegetation. It may be considered a second-line investigation in determining the presence of perivalvular pathology, particularly when aortic valve endocarditis or root abscess is suspected [51]. In the identification of peripheral embolism, scintigraphy with radiolabeled leukocytes or 18-F-fluorodeoxyglucose (FDG) PET/CT increases diagnosis accuracy [52]. Moreover, the latest updates of both ESC/EACTS and ACC/AHA guidelines highlighted the increasing role of PET/CT in the diagnosis of prosthetic valve endocarditis [53, 54].

### Detection of aortic root and vascular access complications

**Fig. 7:**
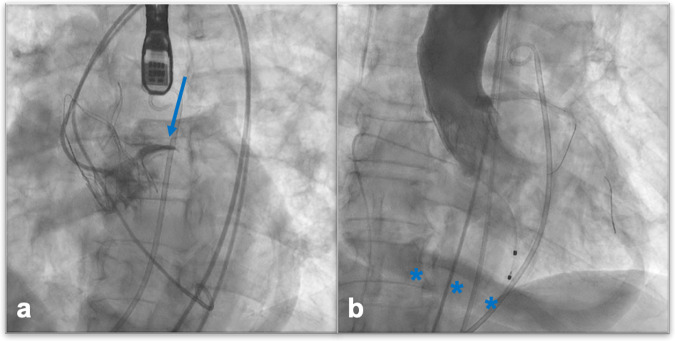
An 85-year-old patient candidate for TAVI for severe symptomatic aortic-stenosis. Intraprocedural death for cardiac arrest was likely due to annular iatrogenic rupture as documented by angiographic images: Iodinated contrast extravasations outside the aorta are demonstrated (arrow, **a**), as well as contrast extravasation into the pericardial sac (asterisks, **b**)

Aortic root complications include a broad pathological spectrum, ranging from mild asymptomatic aortic dilation to life-threatening conditions like aortic dissection and annulus rupture (Fig. 7). Also in this case, some predisposing factors have been identified, like bicuspid aortic valve, kinking of the aorta and calcifications, narrow arch or pre-existing aortic dilation, for which accurate pre-TAVI CTA evaluation is needed [55]. Nearly a quarter of patients exhibit ascending aortic post-TAVI dilatation, which must be documented at post-procedural CT imaging, by correct aortic annulus, bulb, STJ, and ascending tract measurement in oriented MPRs to allow for reproducible measurements at follow-up imaging [56]. Aortic dissection is a relatively rare complication, mostly caused by an intraprocedural unsuccessful attempt to position the prosthetic aortic valve. A dedicated ECG-gated multiphasic CTA scan is central to diagnosing and describing it, further affecting surgical management. Radiological reports must include a careful identification of true and false lumen, with associated intimal flap and eventual involvement of coronary arteries or supra-aortic vessels [57]. Annular rupture during TAVI is a rare but catastrophic event, with mortality rates reaching 50% [58] (Fig. 7). It can result from excessive tissue pressure during various valve deployment steps, particularly when using oversized valves or dealing with heavy calcium buildup. Calcification in the aortic valve, annulus, and LVOT serves as a predictor for annular injury and rupture [59]. Additionally, a meta-analysis has shown an increased incidence of annular rupture in bicuspid versus tricuspid anatomy [60].

**Fig. 8:**
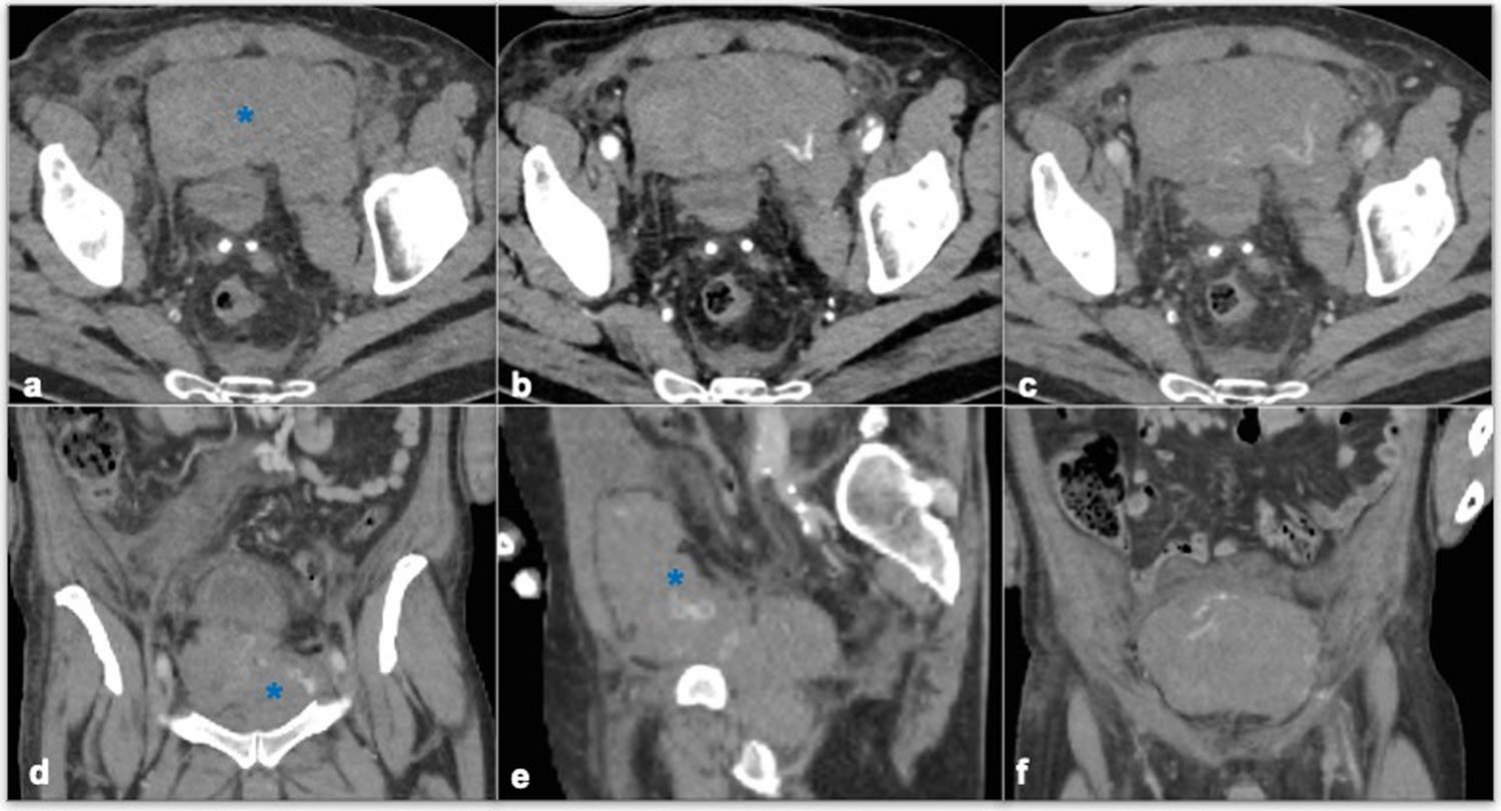
A 70-year-old patient subjected to TAVI 2 days before developed hypotension, anemia (Hb: 9 g/dL) and altered mental status. Contrast-enhanced CTA was performed to rule out acute bleeding, showing a massive lower abdominal spontaneously hyperdense collection (**a**) (asterisk, mean HU = 52), with active bleeding inside (**b**, arrow, arterial phase) (**c**, arrow, venous phase), as a result of an iatrogenic rupture of an arterial femoral-iliac vessel on the left (**d**, **e** asterisks) of 6 × 20 × 8.5 cm (**a**, **f**)

**Fig. 9:**
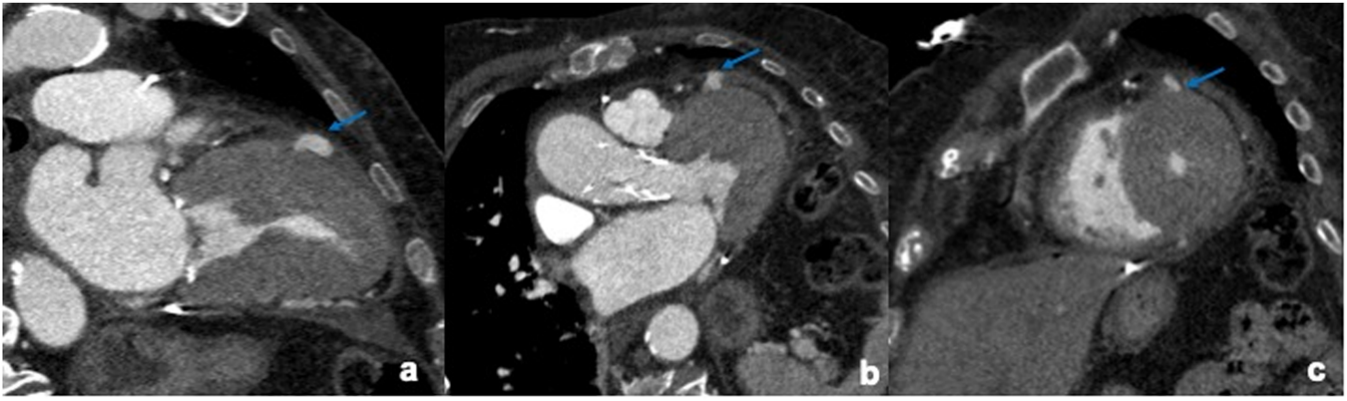
A 55-year-old female patient undergoing post-TAVI CTA for anemia onset (Hb: 9.5 g/dL) after the procedure showing focal blood leakage anteriorly to the left ventricle (**a**, **b**, **c**, arrow) due to an iatrogenic ventricular wall lesion

Vascular access complications (VC) are the most common drawback of TAVI, affecting up to 4-5% of procedures [61]. According to the Valve Academic Research Consortium 3 (VARC) consensus these complications can be classified as vascular and access-site-related complications, whether they affect the vessel itself or involve structures surrounding the access-site [62]. Vascular complications include those occurring acutely during the procedure or at a delayed time (e.g., pseudoaneurysm, AV fistula formation), for which CT represents the modality of choice for diagnosis and follow-up (Fig. 8). They can be attributed to patient-related factors (e.g., female gender, chronic renal failure, iliac-femoral calcifications, and peripheral arterial disease), procedure-related factors (sheath-femoral artery ratio above 1.05 indicating higher risk), and operator and center-related factors (operator experience and institutional expertise) [63]. Closure device failure is also an issue related to VC; different devices can be used in clinical practice like suture-based, collagen, patch, or membrane-based and their failure needs special attention. When they fail to achieve correct hemostasis at the vascular access-site, pseudo-aneurisms and bleeding ensue, implying great morbidity for patients. Pre-TAVI CTA helps guide the access approach by assessing the vascular root, describing vascular dimensions, tortuosity, and plaque assessment [8]. Access-site-related complications, like access-site infection and injuries of the surrounding structures, can be easily assessed by post-procedural CTA. Among them, a special mention is dedicated to trans-apical (TA) approach-related complications. TA strategy is usually preferred for patients with severe vascular diseases, who show higher in-hospital mortality. The main VA-related complication with the TA approach is ventricular tear, possibly leading to massive LV rupture, cardiac tamponade, and death (Fig. 9). CTA is central in detecting these issues, leading to prompt surgical management. Newly designed devices for the TA approach are now decreasing the incidence of these potentially fatal complications, but radiologists’ approach should also concentrate on this kind of LV-related issue when looking at post-TAVI CTA after the TA approach [64].

## Future perspectives and emerging technologies

CTA offers an advanced assessment of myocardial tissue composition, including the measurement of extracellular volume (ECV), which exhibits good agreement with CMR findings [65]. These features have seen significant advancements through the introduction of dual-energy scanners and emerging PCDs, enabling efficient multi-energy imaging and improved tissue differentiation which can be used for the evaluation of aortic-stenosis-associated cardiac amyloidosis. By assessing ECV and characterizing tissue composition, CTA imaging aids in predicting procedural success and guiding therapeutic strategies, offering potentially novel insights into post-TAVI complications [65]. ECV quantified during routine CTA pre-TAVI evaluation can be used to reliably detect amyloid and the measured ECV tracks the degree of infiltration. Besides tissue characterization, CT-derived functional measurements can be valuable in post-TAVI care. Strain imaging, calculated using motion coherence algorithms, provides useful information about myocardial function and early detection of complications like valve thrombosis or bioprosthetic degeneration [66]. Research efforts have explored the potential of CT-derived strain patterns in predicting complications and assessing longitudinal strain changes in patients with severe aortic-stenosis after TAVI. For instance, the effect of pressure overload relief after TAVI on LV apical sparing of longitudinal strain is not well understood. If the LV apical sparing pattern is related to the presence of amyloid protein deposits, it could be hypothesized that the pattern would not change after the procedure [66]. Further research will elucidate the predictive role of CT-derived strain and its clinical significance, offering valuable information for decision-making.

The application of computational fluid dynamics, finite element analysis, and fluid-solid interaction analysis has grown for evaluating valve mechanics and predicting post-TAVI complications. However, their complexity and computational costs have limited their widespread use. Recent advancements in deep learning (DL) provide real-time solutions for rapid hemodynamic parameter assessment to guide treatment selection. DL algorithms have been investigated for various TAVI outcome predictions. For instance, Jia et al achieved an area under the curve (AUC) of 0.84 in predicting late major bleeding after TAVI, outperforming standard models [67]. Agasthi et al [68] used gradient boosting to predict 1-year mortality with an AUC of 0.72, surpassing traditional risk scores. Galli et al [69] combined mechanistic modeling for predicting post-TAVR conduction abnormalities with an AUC of 0.84.

Future research can optimize DL architectures, improve data augmentation techniques, and develop an intelligent DL framework to support personalized prosthetic valve design in real-time, enhancing post-TAVI therapy planning and patient outcomes.

## Conclusion

Post-TAVI complications include a broad spectrum of pathological entities, from asymptomatic and mild to potentially life-threatening conditions. Their recognition is becoming more and more important given the fact that TAVI is now effectively employed in place of SAVR also in intermediate-risk patients. CTA is the mainstay imaging modality for pre-procedural planning of TAVI and is also used for post-interventional early detection of both acute and long-term complications, allowing for detailed morphological analysis of the valve and its movement throughout the entire cardiac cycle. Imaging reliability implies the correct technical setting of the CT scan, knowledge of valve type, normal post-interventional findings, and awareness of classic and life-threatening complications presentation after TAVI. Moreover, novel CTA technologies, like spectral imaging and PCD-CT are opening the possibility of defining new imaging-based markers for post-procedural risk of complication prediction, like ECV and strain, possibly affecting the peri-procedural patients’ management.

## Abbreviations

AR: Aortic regurgitation
AUC: Area under the curve
CT: Computed tomography
CTA: Computed tomography angiography
DL: Deep learning
ECV: Extracellular volume
HALT: hypoattenuating leaflet thickening
HF: Heart failure
IE: Infective endocarditis
LV: Left ventricle
MPR: Multi-planar reconstructions
LVOT: Left ventricular outflow tract
PCD-CT: Photon-counting detector CT
PVL: Paravalvular leakage
SAVR: Surgical aortic valve replacement
STJ: Sino-tubular-junction
SVD: Structural valve degeneration
TA: Trans-apical
TAVI: Transfemoral aortic valve implantation
TEE: Trans-esophageal
TTE: Transthoracic echocardiography
VC: Vascular access complications
ViV: Valve-in-valve
